# Schmallenberg Virus Recurrence, Germany, 2014

**DOI:** 10.3201/eid2107.150180

**Published:** 2015-07

**Authors:** Kerstin Wernike, Bernd Hoffmann, Franz J. Conraths, Martin Beer

**Affiliations:** Friedrich-Loeffler-Institut, Insel Riems, Germany

**Keywords:** Schmallenberg virus, cattle, ruminants, genetics, phylogeny, experimental infection, viruses, Germany, orthobunyavirus, vector-borne infections

## Abstract

Schmallenberg virus (SBV) emerged in Germany in 2011, spread rapidly across Europe, and almost disappeared in 2013. However, since late summer 2014, new cases have occurred in adult cattle. Full-genome analysis revealed some amino acid substitution differences from the first SBV sample. Viremia developed in experimentally infected sheep and cattle for 4–6 days.

Schmallenberg virus (SBV), an insect-transmitted orthobunyavirus with a negative-stranded tripartite RNA genome, causes no or only mild nonspecific clinical signs for a few days in adult ruminants (*1*). However, infection of immunologically naive animals during a vulnerable period of pregnancy can cause premature birth, stillbirth, or severe malformations in the offspring (*2*).

SBV was first detected in autumn 2011 in the blood of an acutely infected cow in North Rhine-Westphalia, Germany (*1*). In early 2012, a large number of malformed lambs and calves, which tested positive for SBV, were born in central Europe (*2*). The malformations resulted from infection of the dams in summer or autumn 2011 and transplacental transmission. Within this first vector season, the virus spread rapidly within and between the animal holdings. In the center of the epidemic in northwestern Germany, the Netherlands, and Belgium, >90% of tested cattle became seropositive (*3*). During the following year, SBV circulated again in Germany (*4*) but at a much lower level and predominantly at the margin of the initially most affected area because susceptible animals had remained there.

During the 2013 vector season and the following winter, SBV cases were detected only sporadically (only SBV genome detections without successful virus isolation) (*5*). For example, only 7 cases of viral genome detection were reported to the German Animal Disease Reporting System during January 1–March 24, 2014; these cases resulted from infection of the respective dams during summer or autumn 2013. The high seroprevalence of the ruminant population and the marked decline of births with SBV-associated malformations in newborns raised hopes that SBV had disappeared after the first epidemic, as occurred with the transient appearance of bluetongue virus serotype 8 in the same region of Europe (*6*).

## The Study

Surprisingly, during summer and autumn 2014, SBV reappeared in Germany to a greater extent. Viral genome was repeatedly detected in acutely infected cattle. Several samples from various federal states were submitted to the Friedrich-Loeffler-Institut, Federal Research Institute for Animal Health (Insel Riems, Germany), to confirm an SBV infection (Table) and to further characterize these reemerging viruses.

**Table:** Origin of Schmallenberg virus real-time reverse transcription PCR–positive samples submitted to the Friedrich-Loeffler-Institut, Germany, 2014

Submission date	Federal state	No. samples	Quantification cycle value
Sep 25	Lower Saxony	3	25.1–28.1
Sep 29	Lower Saxony	1	26.2
Oct 13	North Rhine-Westphalia	1	30.1
Oct 21	Lower Saxony	4	24.1–29.9
Oct 22	North Rhine-Westphalia	2	29.7–30.6
Oct 22	North Rhine-Westphalia	1	31.1
Oct 22	Saxony-Anhalt	1	22.8
Oct 23	Saxony	14	24.1–29.1
Nov 4	Rhineland-Palatinate	4	27.7–38.3
Nov 11	Lower Saxony	1	26.1

To evaluate sequence variations among SBV variants circulating in Germany since 2011, the original SBV isolate (BH80/11) and viruses isolated in 2012 from the blood of viremic sheep (BH619/12) or cattle (D495/12-1 and BH652/12) were compared with 3 genomes obtained from the viruses in acutely infected adult cattle in 2014 (BH119/14-1/2, BH119/14-3/4, BH132/14). RNA was extracted by using the QIAamp Viral RNA Mini Kit (QIAGEN, Hilden, Germany) according to the manufacturer’s recommendations, and the open reading frames of all 3 genome segments (large [L], medium [M], and small [S] segment; primer sequences available on request) were sequenced as described (*7*). Sequences generated in the current study were submitted to GenBank (accession nos. KP731865–KP731882). Sequence alignments and translation in amino acids were supported by Geneious version 7.1 (Biomatters, Auckland, New Zealand). We generated a maximum-likelihood tree (Hasegawa-Kishino-Yano model, 1,000 bootstrap replicates) using MEGA5 (*8*). For the phylogenetic analysis of the M segment, sequences previously obtained from organ samples from newborns malformed because of SBV infection also were integrated (*7*).

For the S segment (830-nt long), which encodes the nucleocapsid protein (N) and a nonstructural protein (NSs), sequence analysis revealed very high stability. The samples obtained from acutely infected animals in 2014, BH619/12 and BH652/12, were 100% identical to the original SBV strain BH80/11 from 2011. In the sample D495/12-1 from 2012, a single nucleotide was substituted (Technical Appendix Figure). The viral RNA-dependent RNA polymerase encoding L segment (6864 nt) also showed high stability, and only a few nucleotide substitutions compared with BH80/11 were found in the latest samples (D495/12-1 and BH652/12: 6 nt; BH619/12: 10 nt; BH119/14-1/2, BH119/14-3/4. and BH132/14: 18 nt). Overall, sequence identity was 99.7%–99.9% (Technical Appendix Figure). Nonsynonymous substitutions ranged from 1 aa to 4 aa (Technical Appendix Figure).

**Figure 1:**
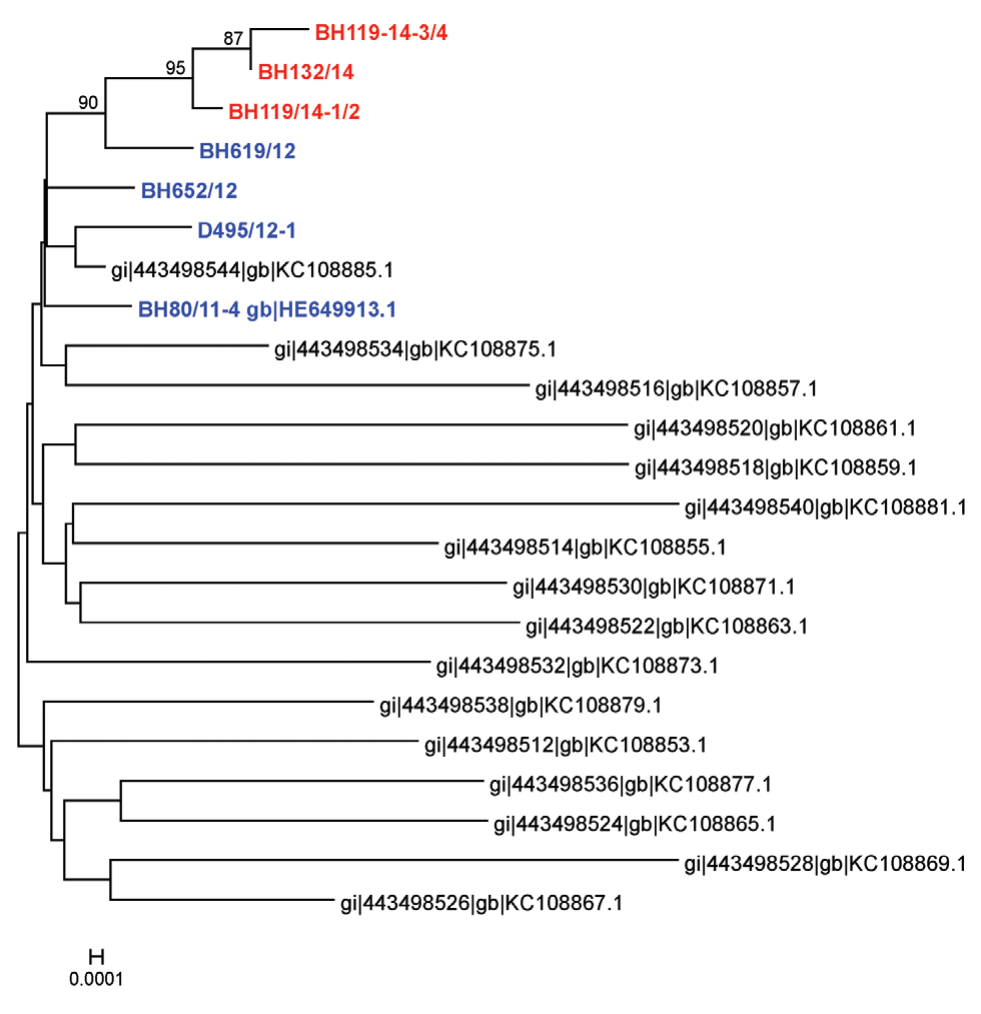
Phylogenetic analysis based on nucleotide sequences of the medium segment of Schmallenberg virus samples isolated from blood of acutely infected animals in 2011 or 2012 (blue) or sequenced directly from the blood of viremic cattle in 2014 (red) and from organ samples of malformed newborns (black) (*7*). Scale bar indicates nucleotide substitutions per site.

The M segment (4415-nt long) encodes 2 glycoproteins (Gn and Gc) and a nonstructural protein (NSm). It is the most variable genome segment of SBV and related viruses (*7*,*9*,*10*). Despite the identification of a highly variable region within the Gc-coding sequence in viruses in malformed newborns (*7*,*9*), there was a high sequence stability of the viruses detected in the blood of acutely infected adult animals; all sequences clustered closely (Figure 1). In contrast to the maximum of 77 nt and 43 aa substitutions or 12 aa deletions or 2 aa insertions found in organ samples of lambs or calves (*7*), we detected only 6–12 nt and 2 aa (BH619/12), 3 aa (D495/12-1, BH119/14-3/4, BH132/14) or 4 aa (BH652/12, BH119/14-1/2) substitutions (Technical Appendix Figure). Because Gn and Gc are major immunogens of orthobunyaviruses (*10*), the mutation hot spot was supposed to be involved in immune evasion mechanisms and/or adaption of the cell tropism within the individual host (*7*,*9*). However, insect-transmitted viruses such as SBV have to adapt to 2 hosts and undergo replication cycles in both the arthropod vector and the mammalian host. Thus, the high sequence stability of virus strains detectable in viremic animals might be necessary for transmission to the vector. Notably, in comparison with the original SBV isolate, K→E substitutions at aa 746 and 1340 of the M segment were found in all other samples. Because these mutations have now been consistently present for at least 2 years, they might have occurred during the adaptation to European ruminants or insects and could provide a growth advantage within the individual host or might be beneficial for transmission between host and vector.

**Figure 2:**
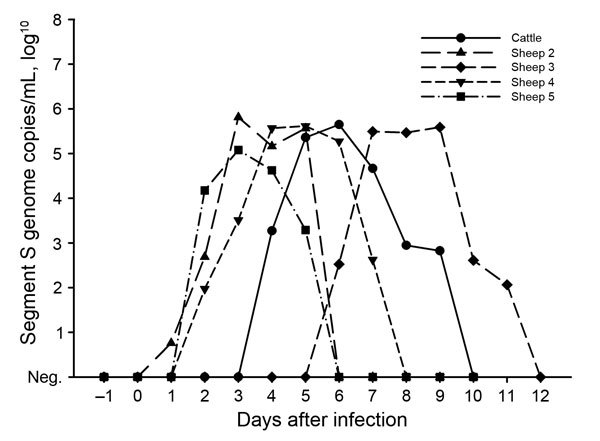
Detection of Schmallenberg virus genome in the blood of experimentally infected cattle and sheep, Germany, 2014.

To investigate whether the detected sequence variations correlated with any change in pathogenicity, 5 female sheep and 1 female calf were subcutaneously inoculated with pools of up to 5 serum or whole blood samples from 1 of the holdings with new cases confirmed in 2014 (sheep 1 and 3 and the calf with samples from Lower Saxony; sheep 2, 4, and 5 with samples from Saxony [permit no. LALLF-M-V/TSD/7221.3–1.1–004/12]). None of the animals showed fever or any other clinical sign. Blood samples were taken daily for the first 2 weeks after inoculation and analyzed by real-time reverse transcription PCR (*11*). Thereafter, serum samples were taken in weekly intervals and tested in a microneutralization assay (*12*) and a commercially available SBV antibody ELISA (ID Screen Schmallenberg Virus Competition; IDvet, Grabels, France). The calf and sheep 2–5 became infected; viral genome was detectable in their blood for 4–6 days (Figure 2), which agrees with the short-lived viremia previously observed after experimental infection of cattle and sheep with the original SBV sample or the first cell culture isolate (*1*,*13*). Antibodies were first detected on day 7 after infection (sheep 2, sheep 5) or day 14 after infection (calf, sheep 3, sheep 4) in both tests.

## Conclusions

SBV, first detected in 2011, circulated again in Germany in 2014. Virus genome with a high sequence identity to the first SBV sample was repeatedly detected in the blood of acutely infected adult cattle, and in experimentally infected animals, viremia developed that was identical to the original SBV isolate. The renewed virus circulation during the 2014 vector season was observed primarily in an area less affected in the 2 previous years. The missing or markedly reduced virus circulation led to a decline in herd seroprevalence caused by a missing infection of the young stock (*14*,*15*); further reasons for the unexpected recurrence of SBV could be persistence within the insect vectors. As a consequence, the infection of naive animals in autumn 2014 resulted in an increasing frequency of the birth of malformed offspring in the following winter.

**Technical Appendix.** Comparison of the small-, medium-, and large-segment sequences of Schmallenberg viruses isolated in 2012 from the blood of viremic ruminants or detected in 2014 in acutely infected adult cattle with the first SBV isolate from 2011, Germany.
