# Supplementary material for: Schmallenberg Virus Recurrence, Germany, 2014
Source: Emerg Infect Dis. 2015 Jul;21(7):1202–4. doi: 10.3201/eid2107.150180 (PMC4480399; doi:10.3201/eid2107.150180)
Supplement: Supplementary file 1 — Technical Appendix. Comparison of the small-, medium-, and large-segment sequences of Schmallenberg viruses isolated in 2012 from the blood of viremic ruminants or detected in 2014 in acutely infected adult cattle with the first SBV isolate from 2011, Germany. [file 15-0180-Techapp-s1.pdf]

Article DOI: http://dx.doi.org/10.3201/eid2107.150180

Schmallenberg Virus Recurrence, Germany, 2014

Technical Appendix

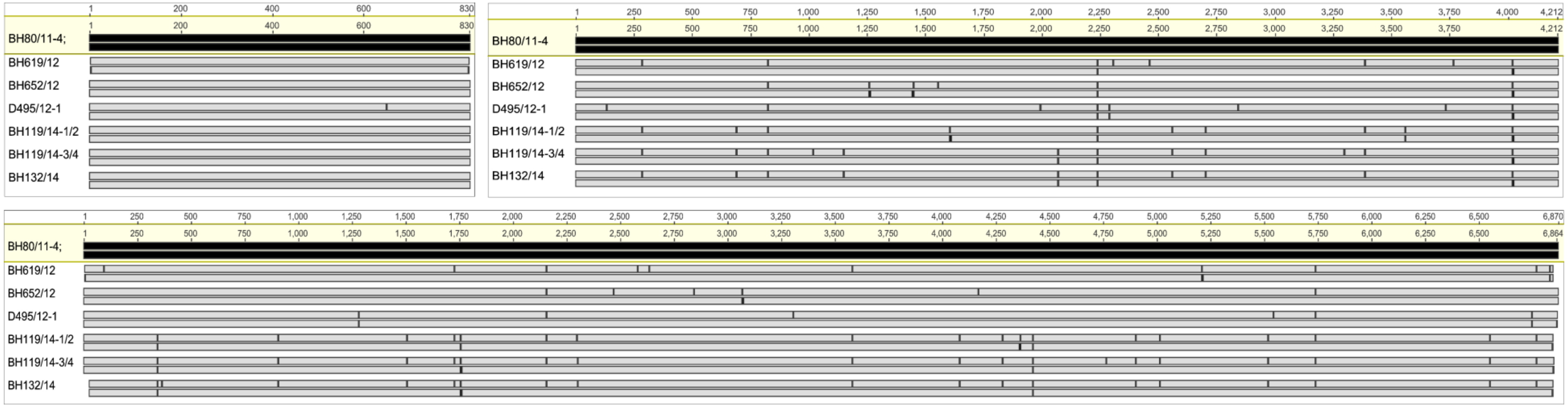

Technical Appendix Figure. Comparison of the S- (upper left image), M- (top right), and L-segment sequences (bottom) of SBVs isolated in 2012 from the blood of viremic ruminants or detected in 2014 in acutely infected adult cattle with the first SBV isolate from 2011 (shaded in yellow) in Germany; nucleotide (upper gray bar alongside the name of each isolate) and amino acid (lower gray bar) substitutions are highlighted as vertical black lines. L, large; M, medium; S, small; SBV, Schmallenberg virus.
